# Twenty-Four-Hour Ambulatory Blood Pressure Variability Associated With Cerebral Small Vessel Disease MRI Burden and Its Progression in Inpatients With Cerebrovascular Disease

**DOI:** 10.3389/fneur.2020.513067

**Published:** 2020-09-30

**Authors:** Yangyi Fan, Chang Hou, Li Peng, Xuguang Gao, Yan Xu

**Affiliations:** ^1^Department of Neurology, Peking University People's Hospital, Beijing, China; ^2^Department of Cardiology, Peking University People's Hospital, Beijing, China

**Keywords:** ambulatory blood pressure monitoring (ABPM), blood pressure variability, cerebral small vessel disease, brain magnetic resonance image (MRI), cerebrovascular disease

## Abstract

**Background:** Lacunar infarcts, white matter lesions, cerebral microbleed, enlarged perivascular space and brain atrophy are regarded as magnetic resonance imaging (MRI) manifestations of cerebral small vessel disease (cSVD). 24-hour blood pressure variability (BPV) has been reported to relate with cerebral small vessel disease, but the impact of 24-h BPV on the total MRI cSVD burden and its progression in inpatients with cerebrovascular disease has not been investigated yet.

**Methods:** We enrolled inpatients with cerebrovascular disease, who underwent the 24-h ambulatory blood pressure monitoring (ABPM) and the brain MRI scan at baseline and had the follow-up brain MRI images stored in the clinical information system of our hospital. BPV was quantified by the calculation of standard deviation (SD), coefficient of variation (CV), weighted standard deviation (wSD) of blood pressure record. We evaluated the total cSVD score on baseline MRI and the MRI followed-up to obtain the total burden of cSVD. The cSVD burden progression was estimated through the comparison of the total cSVD score on the two MRIs.

**Results:** A total of 140 patients with an average age of 65.6 years were finally enrolled, 82.9% (116/140) of whom had one or more cSVD markers. After a median of 4.4 years follow-up, cSVD score progression were found in 50.7% (71/140) of the patients. Both SD and CV of SBP and DBP during 24-h and daytime as well as the SBP wSD differed significantly among different total cSVD score groups. The SBP SD and CV during 24-h and daytime, the SBP SD in nighttime, the DBP SD and CV during the daytime were significantly higher in the cSVD progression group than those in the cSVD no-progression group. The SBP wSD and the DBP wSD were significantly higher in the cSVD progression group than those in the cSVD no-progression group. Logistic regression analyses revealed that daytime SBP SD and SBP wSD were independent risk factors for total cSVD burden [daytime SBP SD: OR = 1.628, 95% CI = 1.105–2.398 (per 5 mmHg increase in SD), *P* = 0.014; SBP wSD: OR = 2.248, 95% CI = 1.564–3.230 (per 5 mmHg increase in wSD), *P* < 0.001)] and SBP wSD was a significant predictor for cSVD progression [OR = 2.990, 95% CI = 1.053–8.496 (per 5 mmHg increase in wSD), *P* = 0.040].

**Conclusion:** Higher BPV were significantly related with total cSVD burden in inpatients with cerebrovascular disease. SBP SD during daytime and SBP wSD were independent risk factor for total cSVD burden and SBP wSD was an predictive factor for cSVD progression.

## Introduction

Cerebral small vessel disease (cSVD) is a progressive syndrome which involves the perforating arterioles, capillaries and venules of the brain (1). It often manifests as stroke, cognitive impairment, gait disturbance and urination disorder, and accounts for 20–25% of strokes and 45% of dementias in the elderly (2). Up to date, six neuroimages have been acknowledged as markers of cSVD on magnetic resonance imaging (MRI): recent small subcortical infarct, lacunar infarcts, white matter lesions, cerebral microbleed, perivascular space enlargement and brain atrophy (3), and the total burden of cSVD can be assessed by the total cSVD score including the markers above (4, 5). The etiology and pathogenesis of cSVD have not yet been completely understood, but classical vascular risk factors such as age, hypertension, diabetes are considered to relate to cSVD (6–8).

Previous studies have confirmed that 24-h ambulatory blood pressure monitoring (ABPM) is more useful to predict the hypertension-related cardiovascular risk than casual clinical blood pressure measurement (9, 10), and the 24-h blood pressure variability (BPV) documented by ABPM has been revealed an independent risk factor of the target organ damage caused by hypertension (11). Recent studies have found increased BPV correlates with white matter lesions, microbleed and enlarged perivascular space in the brain (12–14). The relationship of BPV and the total burden of cSVD has also been explored in some specific population lately (4, 5, 15), but the impact of BPV on the total cSVD burden has not yet been investigated in inpatients with cerebrovascular disease. Besides, there is also lack of studies investigating the relationship of baseline BPV and the cSVD progression longitudinally. For patients with cerebrovascular disease usually have more risk factors mentioned above and have a higher risk of cSVD development than normal population, we aim to explore the impact of BPV on total cSVD burden and cSVD progression in inpatients with cerebrovascular disease.

## Materials and Methods

### Subjects

We retrospectively recruited the inpatients with cerebrovascular disease in the Neurology Department of Peking University People's Hospital from August 1, 2011 to October 31, 2012, who underwent the 24-h ABPM and brain MRI within 14 days after the admission as well as the reexamination of brain MRI before January 31, 2019. All the patients were managed in our stroke clinic and recorded in the clinical information system of our hospital. The following patients were excluded: (1) patients with previous severe brain trauma, infectious and toxic brain injury; (2) patients with past or acute severe ischaemic or haemorrhagic stroke (because of the difficulty in MRI assessment for cSVD); (3) patients with acute myocardial infarction, atrial fibrillation or other severe heart disease such as congestive heart failure, severe nephritic or hepatic insufficiency, tumor; (4) patients who cannot afford the ideal MRI imaging or valid 24-h ABPM recording for the assessment (the criteria for invalid was described below).

The diagnosis of cerebrovascular disease was made according to the diagnosis and classification of ICD-10 (16). The following clinical information were recorded: age, sex, body mass index (BMI), history of hypertension, diabetes mellitus, smoking, hypercholesterolemia and previous stroke. Laboratory tests including uric acid, total cholesterol, triglycerides, high-density lipoprotein cholesterol, low-density lipoprotein cholesterol, glycosylated hemoglobin (HbA1c) were also recorded.

### Twenty-Four-Hour ABPM Recording

Twenty-four-hour ABPM was performed for all the enrolled patients within 14 days after admitted to hospital, using an automatic ambulatory blood pressure recorder (90217, Spacelabs, America). The instruments were set to record blood pressure every 30 min in the daytime (6:00–22:00) and every 1 h in the nighttime (22:00-next 6:00). The recordings with less than 70% measurements or 2 measurements per hour in daytime or 1 measurement per hour in nighttime were considered to be invalid. The mean systolic blood pressure (SBP), diastolic blood pressure (DBP) as well as the corresponding standard deviation (SD) during daytime, nighttime and 24 h were collected. The SD and coefficient of variation (CV) of SBP and DBP during daytime, nighttime and 24 h were chosen to be metrics of short-term BPV. The weighted SD (wSD) of SBP and DBP in 24 h, which were considered better BPV metrics for they remove the interference of nocturnal blood pressure fall, were also used. The CV was defined as the ratio of the SD and the mean SBP or DBP at the same periods. The wSD was calculated according to the following formula.

$$\begin{array}{l} weight\;SD=\frac{daytime\;SD\times T_{day}+nighttime\;SD\times T_{night}}{T_{total}} \end{array}$$

### MRI Assessments

Brain MRI was performed for the patients within 14 days after admission, using a 1.5 T or 3.0 T scanner (GE 750 or GE 750W, America). The sequences of MRI included the T1-weighted, T2-weighted, diffusion-weighted, fluid attenuated inversion recovery (FLAIR) and susceptibility weighted imaging (SWI). The images were independently assessed by two neurologic radiologists who were blind to the clinical information and to each other's reading. If there was a divergence, a consultation would be held to reach an agreement.

The total cSVD score grading from 0 to 5 was assessed using the method reported previously (4, 5, 15) according to the presence of the following five MRI markers of cSVD, which reflected the total burden of cSVD.

1. Lacunar infarcts: We defined as lesions with cerebrospinal fluid-like signal on all sequences, and with a hyperintense rim surrounding the lesion. The diameter of the lesion should be between 3 and 20 mm, and its location should be in the territory of a perforating arteriole, such as the basal ganglia, thalamus, internal or external capsule, or the brain stem (2). If there was one or more lacunar infarct, one point was awarded.
2. White matter hyperintensity (WMH): WMH was assessed using the Fazekas scale on FLARI (17). If periventricular WMH Fazekas score reached 3 or deep WMH Fazekas score was 2 or 3, one point was awarded.
3. Enlarged perivascular spaces (EPVS): We defined EPVS as cerebrospinal fluid-like lesions with an ovoid, round, or linear shape and a diameter <3 mm. We counted the number of EPVS at the level of the basal ganglia because former studies showed EPVS at this level seemed associated with cSVD more specifically. Then we chose the slide with the highest number in one hemisphere If the number was more than 10, one point was awarded (18, 19).
4. Microbleed: Because former literatures revealed that deep microbleeds related with cSVD, whereas lobar microbleeds related to a great extent with amyloid angiopahty, we awarded one point to the total score only if one or more microbleeds were found in the deep area on SWI (20, 21).
5. Brain atrophy: We evaluated brain atrophy according to the visual rating scale of Pasquier et al. and Victoroff et al. (22, 23). If there was moderate-extensive brain atrophy, one point was awarded.

The latest images of the MRI saved in the clinical information system were also reevaluated with the same process and criteria above. If the total cSVD score was larger than that at baseline, we adjudicated the patient with cSVD progression.

### Statistical Analysis

Statistical analysis was performed with the SPSS 19.0 (IBM Corp., Armonk, NY) and the difference was considered statistically significant if *P* < 0.05. Data of continuous variables were presented as mean ± SD if normally distributed and median (interquartile range, IQR) otherwise. Analysis of variance and independent *t*-test was used for the comparison among groups for the normally distributed continuous variables, while Kruskal-Wallis test was used if the variables were abnormally distributed. Date of categorical variables were presented as n (%), and χ^2^ test was used for determining the difference between groups. Spearman correlation analysis was performed to examine the correlation of blood pressure and BPV metrics with total cSVD score. Ordinal logistic regression was performed to explore whether the metrics of BPV were independent risk factors for the burden and binary logistic regression for the progression of cSVD, adjusted for the classical vascular risk factors such as sex, age, diabetes, smoking, hyperlipidemia, BMI, the SBP and DBP level.

## Results

**Figure 1:**
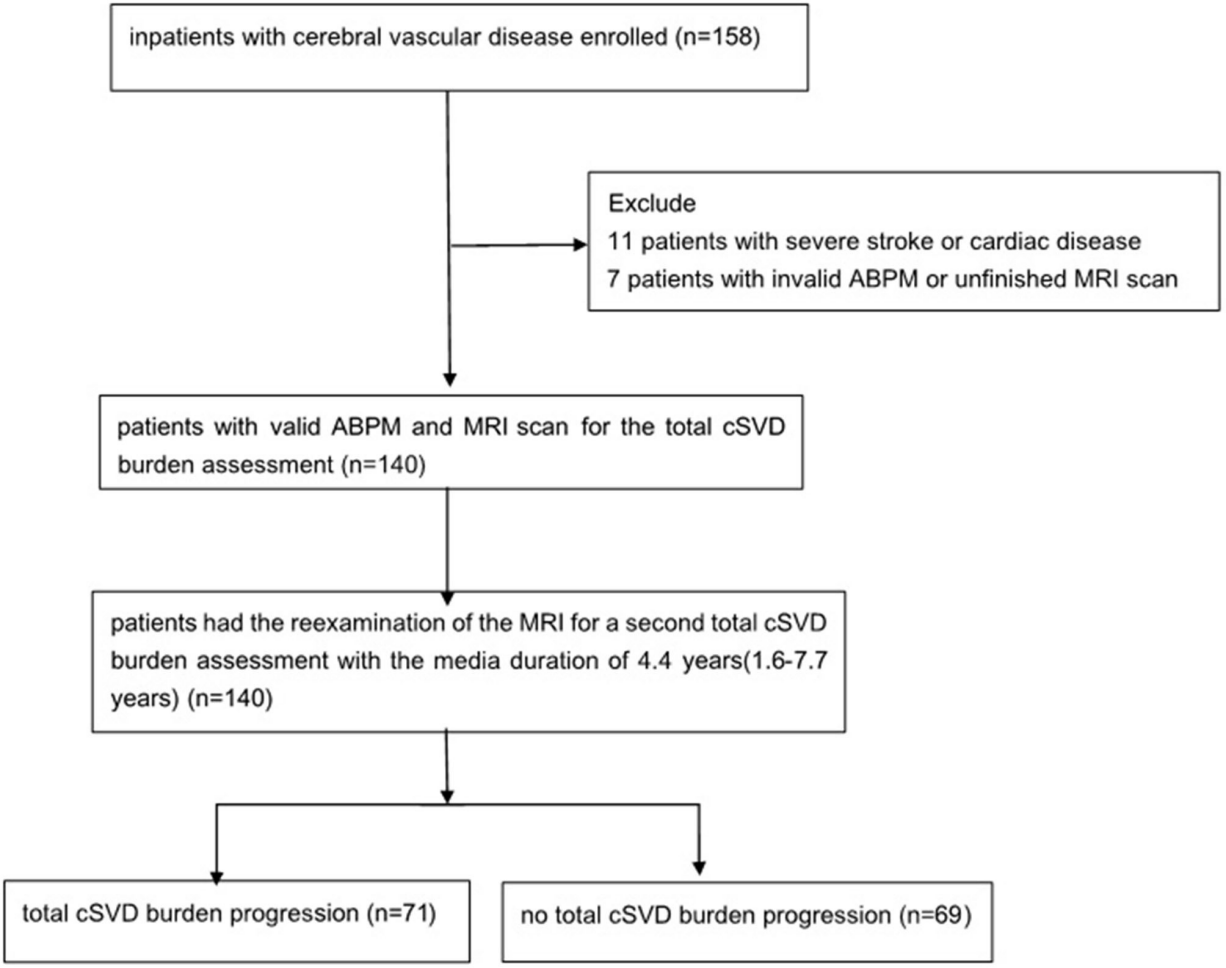
Study flowchart. ABPM, ambulatory blood pressure monitoring; MRI, magnetic resonance imaging; cSVD, cerebral small vessel disease.

**Figure 2:**
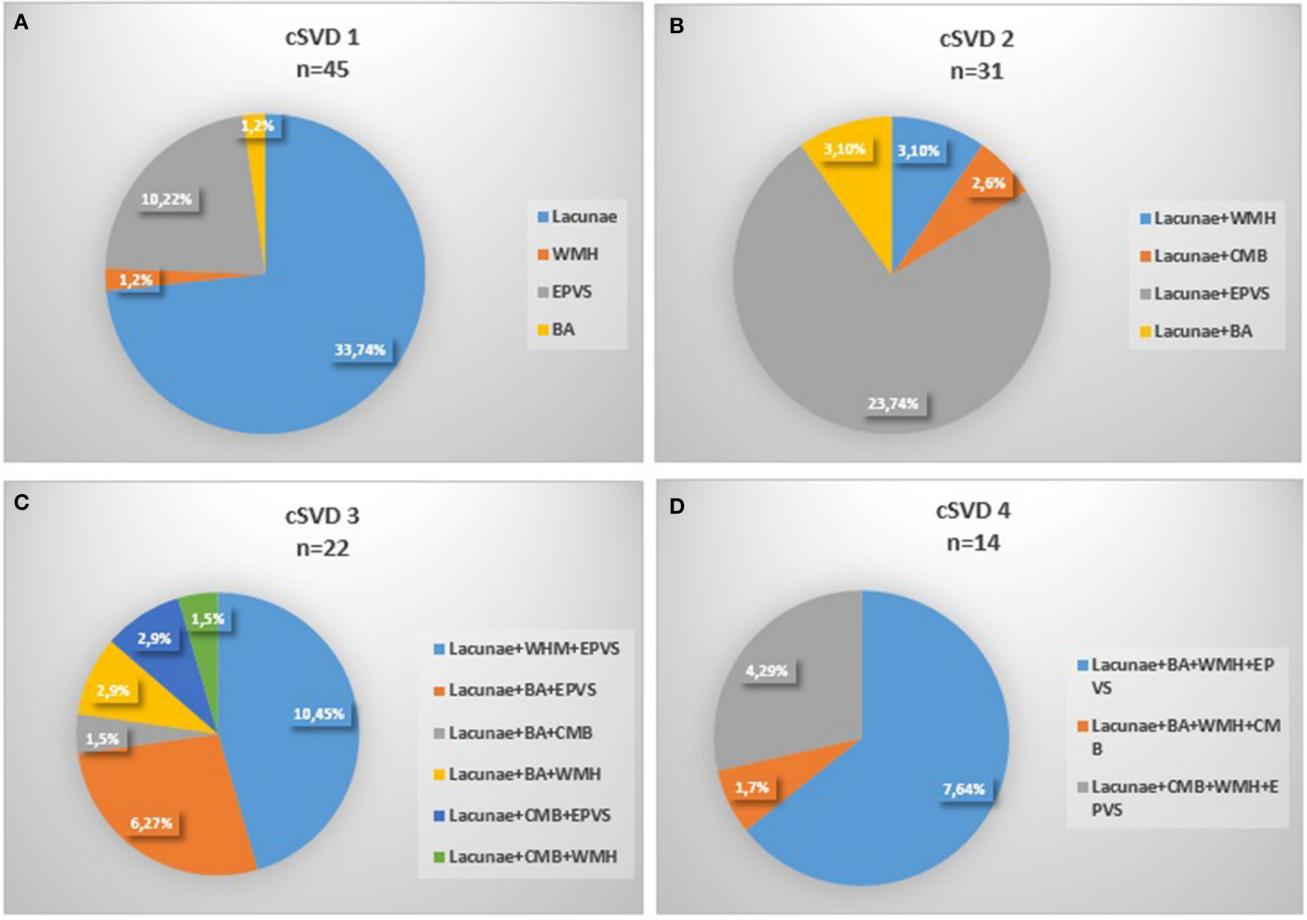
Composition of the different categories of cSVD scale. **(A)** 1 marker of cSVD, **(B)** 2 markers of cSVD, **(C)** 3 markers of cSVD, **(D)** 4 markesr of cSVD, 0 and 4 markers were not shown for none or all markers existed. cSVD, cerebral small vessel disease; BA, brain atrophy; CMB, cerebral microbleed; EPVS:,enlarged perivascular space; WMH, white matter hyperintensity.

One hundred and fifty-eight patients in the Neurology Department of Peking University People's Hospital were enrolled with the time of admition from August 1, 2011 to October 31, 2012. Eleven patients were excluded because of severe stroke or cardiac disease, 7 patients were excluded for the invalid ABPM data or unfinished MRI scan, and finally 140 patients were enrolled (Figure 1). The mean age was 65.6 ± 12.4 years old and 67.1% (94/140) of them were male. With regard to the major initial diagnosis, 41.4% (58/140) of the patients were diagnosed with acute cerebral infarction, 20.0% (28/140) diagnosed with old infarcts, 8.6% (12/140) diagnosed with transient ischemic attack (TIA), and 30.0% (42/140) diagnosed with posterior circulation ischemia. In terms of the cSVD burden, 45 (32.1%) of the patients had 1 marker, whereas 24 (17.1%) patients had no markers and 4 (2.9%) patients had all the five markers. The composition of categories for each cSVD score was showed in Figure 2. For there were only 4 patients having all the five markers, we finally divided all the patients into five groups with the cSVD score grading from 0 to ≥ 4.

Age differed significantly among five groups, with higher ages in groups having more cSVD markers (*P* < 0.001) and significant higher percentage of previous stroke was found in greater total cSVD score groups (*P* = 0.045). Sex, smoking, history of hyperlipidemia or diabetes mellitus did not differ significantly among different total cSVD score groups. There was a tendency of larger proportion of hypertension with the total cSVD score increasing, but the difference was not statistically significant. No significant difference was observed in the level of BMI, HbA1c, uric acid, or lipid profiles including total cholesterol, triglycerides, high-density lipoprotein and low-density lipoprotein cholesterol (Table 1).

**Table 1:** Demographic and clinical characteristics of patients in different cSVD burden groups.

**Total cSVD score**	**0**	**1**	**2**	**3**	**≥ 4**	***P***
	**(*n* = 24)**	**(*n* = 45)**	**(*n* = 31)**	**(*n* = 22)**	**(*n* = 18)**	
Age (years)	58.7 ± 11.6	60.6 ± 12.4	68.6 ± 10.8	71.0 ± 10.3	75.4 ± 7.1	<0.001
Sex (male), *n* (%)	17 (70.8)	27 (60.0)	21 (67.7)	15 (68.2)	14 (77.8)	0.712
Previous stroke, *n* (%)	2 (8.3)	10 (22.2)	9 (29.0)	9 (40.9)	8 (44.4)	0.045
Hypertension, *n* (%)	16 (66.7)	29 (64.4)	27 (87.1)	20 (90.9)	14 (77.8)	0.057
Diabetes, *n* (%)	7 (29.2)	13 (28.9)	11 (35.5)	4 (18.2)	4 (22.2)	0.690
Hyperlipidemia, *n* (%)	1 (8.3)	8 (18.7)	4 (9.8)	1 (4.5)	1 (5.6)	0.417
Smoking, *n* (%)	9 (37.5)	16 (35.6)	9 (29.0)	7 (31.8)	6 (33.3)	0.967
BMI (kg/m^2^)	25.0 ± 3.2	25.3 ± 3.3	24.5 ± 3.8	25.4 ± 3.8	24.8 ± 3.2	0.830
Total cholesterol, mmol/L	4.3 ± 0.9	4.6 ± 1.0	4.3 ± 0.8	4.3 ± 1.1	4.0 ± 1.1	0.338
Triglycerides, mmol/L	1.3 (0.9–2.1)	1.3 (1.1–1.8)	1.2 (0.9–1.6)	1.4 (1.1–1.7)	1.2 (0.9–1.6)	0.613
High-density lipoprotein, mmol/L	1.1 ± 0.3	1.1 ± 0.3	1.1 ± 0.3	1.0 ± 0.2	0.9 ± 0.2	0.068
Low-density lipoprotein, mmol/L	2.7 ± 0.7	2.8 ± 0.9	2.6 ± 0.8	2.7 ± 1.0	2.4 ± 0.9	0.673
HbA1c, %	6.3 (5.7–7.3)	6.2 (5.8–7.2)	6.5 (5.8–7.6)	5.8 (5.7–6.5)	6 (5.7–7.5)	0.320
Uric acid	327.7 ± 77.7	316.6 ± 89.7	297.2 ± 88.3	357.0 ± 75.2	354.1 ± 86.5	0.071

*cSVD, cerebral small vessel disease; BMI, body mass index; HbA1c, glycosylated hemoglobin*.

### Relation Between 24-h ABP Levels, BPV, and the Burden of cSVD

SBP levels increased significantly with higher total cSVD score during 24-h (*P* = 0.018), daytime (*P* = 0.018) and nighttime (*P* = 0.035) (Table 2). The Spearman correlation analysis revealed significantly positive relation between SBP levels and total cSVD score, however, there was not significant relationship between DBP levels and total cSVD score (Table 3).

**Table 2:** Blood pressure levels and variability in different cSVD burden groups.

**Total cSVD score**	**0**	**1**	**2**	**3**	**≥ 4**	***P***
	**(*n* = 24)**	**(*n* = 45)**	**(*n* = 31)**	**(*n* = 22)**	**(*n* = 18)**	
**24 h**						
SBP, mmHg	122.3 ± 12.3	124.1 ± 14.7	129.2 ± 11.3	132.5 ± 17.9	134.2 ± 17.3	0.018
DBP, mmHg	75.6 ± 9.5	75.1 ± 10.1	75.0 ± 9.2	76.5 ± 8.2	73.4 ± 7.5	0.875
SBP SD, mmHg	10.3 ± 3.3	10.9 ± 2.5	12.6 ± 3.0	14.5 ± 3.2	15.0 ± 3.2	<0.001
DBP SD, mmHg	7.5 ± 1.9	8.0 ± 1.6	9.0 ± 2.4	10.1 ± 2.6	9.9 ± 3.6	<0.001
SBP CV, %	8.4 ± 2.7	8.9 ± 2.0	9.8 ± 2.5	11.0 ± 2.5	11.3 ± 2.4	<0.001
DBP CV, %	10.1 ± 2.8	10.7 ± 2.5	12.3 ± 4.0	13.3 ± 3.5	13.6 ± 5.3	0.002
SBP wSD, mmHg	9.2 ± 2.6	9.8 ± 2.2	11.5 ± 2.9	13.7 ± 3.1	13.2 ± 3.0	<0.001
DBP wSD, mmHg	9.8 ± 3.9	10.0 ± 3.6	10.8 ± 4.1	11.1 ± 4.5	11.0 ± 3.3	0.646
**Daytime (6:00–22:00)**						
SBP, mmHg	123.9 ± 12.2	125.8 ± 14.2	130.2 ± 12.4	133.6 ± 17.5	136.4 ± 16.9	0.018
DBP, mmHg	76.6 ± 9.1	76.5 ± 9.9	75.9 ± 9.5	78.3 ± 9.1	75.5 ± 7.2	0.886
SBP SD, mmHg	9.7 ± 3.0	10.4 ± 2.6	12.2 ± 3.2	14.7 ± 3.5	14.2 ± 2.9	<0.001
DBP SD, mmHg	7.1 ± 1.7	7.4 ± 1.7	8.6 ± 2.7	9.7 ± 2.2	9.5 ± 4.1	<0.001
SBP CV, %	7.8 ± 2.2	8.2 ± 1.9	9.5 ± 2.6	11.1 ± 2.6	10.4 ± 1.9	<0.001
DBP CV, %	9.4 ± 2.3	9.8 ± 2.5	11.6 ± 4.3	12.6 ± 3.2	12.6 ± 5.7	0.001
**Nighttime (22:00–6:00)**						
SBP, mmHg	118.9 ± 14.7	120.0 ± 16.5	126.9 ± 11.2	130.3 ± 20.1	129.4 ± 21.8	0.035
DBP, mmHg	72.9 ± 11.9	71.8 ± 10.9	72.9 ± 9.8	74.9 ± 9.6	69.0 ± 9.5	0.498
SBP SD, mmHg	10.3 ± 4.4	10.4 ± 4.1	11.2 ± 4.8	11.4 ± 4.7	11.7 ± 3.7	0.727
DBP SD, mmHg	7.8 ± 2.5	8.3 ± 2.6	9.2 ± 3.4	9.9 ± 4.3	7.9 ± 2.7	0.094
SBP CV, %	8.6 ± 3.2	8.8 ± 3.5	8.9 ± 4.0	8.9 ± 3.7	9.2 ± 3.2	0.990
DBP CV, %	11.0 ± 4.0	11.8 ± 4.3	12.8 ± 4.9	13.6 ± 5.9	11.7 ± 4.4	0.368

*cSVD, cerebral small vessel disease; SBP, systolic blood pressure; DBP, diastolic blood pressure; SD, standard deviation; CV, coefficient of variation; wSD, weighted standard deviation*.

**Table 3:** Correlation analysis of total cSVD burden and BP, BPV.

**BP and BPV metrics**	***r***	***P***
**24 h**		
SBP	0.278	0.001
DBP	0.017	0.843
SBP SD	0.278	0.001
DBP SD	0.362	<0.001
SBP CV	0.418	<0.001
DBP CV	0.321	<0.001
SBP wSD	0.525	<0.001
DBP wSD	0.147	0.040
**Daytime (6:00–22:00)**		
SBP	0.270	0.001
DBP	0.015	0.865
SBP SD	0.524	<0.001
DBP SD	0.365	<0.001
SBP CV	0.454	<0.001
DBP CV	0.360	<0.001
**Nighttime (22:00–6:00)**		
SBP	0.233	0.006
DBP	0.010	0.903
SBP SD	0.156	0.067
DBP SD	0.101	0.238
SBP CV	0.037	0.664
DBP CV	0.084	0.325

*cSVD, cerebral small vessel disease; SBP, systolic blood pressure; DBP, diastolic blood pressure; SD, standard deviation; CV, coefficient of variation; wSD, weighted standard deviation*.

The SD and CV of both SBP and DBP during 24-h and daytime differed significantly among the five groups, with greater SD and CV in higher total cSVD score groups, but we did not find significant difference in nighttime BPV metrics (SD, CV). The SBP wSD was also significantly different among subjects with different cSVD scores but the DBP wSD was not (Table 2). The Spearman correlation analysis demonstrated higher SD and CV of both SBP and DBP in 24-h and daytime correlated with higher total cSVD score significantly (Table 3). Ordinal logistic regression analysis indicated that SBP SD in daytime was an independent risk factor for total cSVD score after adjusting for age, sex, BP levels, the history of previous stroke and other conventional risk factors (Table 4). When wSD was used as the factors calculating BPV, both the SBP and the DBP wSD were found significant correlation with the total cSVD burden (Table 3). In the ordinal logistic regression, SBP wSD was showed an independent risk factor for total cSVD score even after adjusting the factors mentioned above (Table 4), while the DBP wSD was not.

**Table 4:** Daytime SBP SD in relation to total cSVD burden by ordinal regression analysis.

	**Model 1**		**Model 2**		**Model 3**	
	**OR (95% CI)**	***P***	**OR (95% CI)**	***P***	**OR (95% CI)**	***P***
Daytime SBP SD, mmHg	3.410 (1.689–6.883)	0.001	2.954 (1.405–6.221)	0.004	1.628 (1.105–2.398)	0.014
SBP wSD mmHg	2.889 (1.970–4.238)	<0.001	2.256 (1.549–3.286)	<0.001	2.248 (1.564–3.230)	<0.001

*Model 1: adjusted for BP levels during the same time period; Model 2: Model 1+ age and sex; Model 3: Model 2+previous stroke, smoking, diabetes mellitus, hyperlipidemia, BMI. Results of the ordinal logistic regression were presented as OR per 5 mmHg increase in SBP SD and OR per 5 mmHg increase in SBP wSD*.

*cSVD, cerebral small vessel disease; SBP, systolic blood pressure; SD, standard deviation; wSD, weighted standard deviation; BMI, body mass index*.

### Relation Between 24-h ABP Levels, BPV, and the cSVD Burden Progression

The duration between the latest MRI and the baseline ranged from 1.6 to 7.7 years and the median duration was 4.4 years. Among the 140 patients, 71 (50.7%) patients were found total cSVD burden progression. Compared with those without total cSVD burden progression, the patients with progression were significantly older (*P* < 0.001) and had higher baseline total cSVD score (*P* = 0.002). The percentage of hypertension (*P* = 0.004) and the levels of HbA1c (*P* = 0.045) was significantly higher in the total cSVD burden progression group. The difference in other characteristics including sex, the years of follow-up, history of smoking, diabetes, hyperlipidemia was not significant between the subjects with or without total cSVD burden progression. Besides, BMI, serum lipid and uric acid were comparable between two groups (Table 5).

**Table 5:** The baseline characteristics of patients with or without cSVD burden progression.

**cSVD burden progression**	**No**	**Yes**	***P***
	**(*n* = 69)**	**(*n* = 71)**	
Age (years)	61.6 ± 12.7	69.4 ± 10.9	<0.001
Sex (male), *n* (%)	48 (69.6)	46 (64.8)	0.547
Baseline cSVD score	1 (0–2)	2 (1–3)	0.002
Years of follow-up	4.3 (3.4–5.3)	4.5 (3.4–5.7)	0.405
Hypertension, *n* (%)	45 (65.2)	61 (85.9)	0.004
Diabetes, *n* (%)	14 (20.3)	25 (35.2)	0.072
Hyperlipidemia, *n* (%)	6 (8.7)	11 (15.5)	0.218
Smoking, *n* (%)	28 (40.6)	19 (26.8)	0.083
BMI (kg/m^2^)	25.2 ± 3.2	24.9 ± 3.7	0.583
Total cholesterol, mmol/L	4.4 ± 1.0	4.4 ± 1.0	0.916
Triglycerides, mmol/L	1.3 (1.0–1.8)	1.3 (1.0–1.7)	0.738
High-density lipoprotein, mmol/L	1.1 ± 0.3	1.1 ± 0.3	0.927
Low-density lipoprotein, mmol/L	2.7 ± 0.9	2.6 ± 0.9	0.906
HbA1c, %	6.0 (5.7–6.5)	6.4 (5.8–7.1)	0.045
Uric acid	316.6 ± 80.3	334.2 ± 91.9	0.233
**24-h**			
SBP, mmHg	123.2 ± 13.5	131.7 ± 15.3	0.001
DBP, mmHg	75.7 ± 9.5	74.7 ± 8.8	0.493
SBP SD, mmHg	11.0 ± 3.4	13.6 ± 3.0	<0.001
DBP SD, mmHg	8.3 ± 1.9	9.2 ± 2.9	0.052
SBP CV, %	9.0 ± 2.8	10.3 ± 2.1	0.001
DBP CV, %	11.2 ± 3.3	12.4 ± 4.0	0.054
SBP wSD, mmHg	9.5 ± 2.7	11.7 ± 2.8	<0.001
DBP wSD, mmHg	9.4 ± 3.7	10.8 ± 3.5	0.024
**Daytime (6:00–22:00)**			
SBP, mmHg	124.7 ± 13.1	133.2 ± 15.3	0.001
DBP, mmHg	77.2 ± 9.3	75.9 ± 9.0	0.418
SBP SD, mmHg	10.4 ± 3.3	13.2 ± 3.1	<0.001
DBP SD, mmHg	7.7 ± 1.8	8.8 ± 3.1	0.016
SBP CV, %	8.4 ± 2.5	10.0 ± 2.3	<0.001
DBP CV, %	10.2 ± 3.0	11.6 ± 4.3	0.025
**Nighttime (22:00–6:00)**			
SBP, mmHg	119.6 ± 16.0	128.6 ± 11.1	0.002
DBP, mmHg	73.0 ± 11.5	71.8 ± 9.5	0.498
SBP SD, mmHg	10.0 ± 4.4	11.8 ± 4.1	0.016
DBP SD, mmHg	8.5 ± 3.1	8.7 ± 3.2	0.678
SBP CV, %	8.5 ± 3.8	9.2 ± 3.2	0.262
DBP CV, %	12.0 ± 5.0	12.3 ± 4.3	0.739

*cSVD, cerebral small vessel disease; BMI, body mass index; HbA1c, glycosylated hemoglobin; SBP, systolic blood pressure; DBP, diastolic blood pressure; SD, standard deviation; CV, coefficient of variation; wSD, weighted standard deviation*.

SBP levels of the total cSVD burden progression group during 24-h (*P* = 0.001), daytime (*P* = 0.001) and nighttime (*P* = 0.002) were all significantly higher, but there was no significant difference in DBP levels between two groups. The SBP SD and CV during 24-h and daytime as well as the SD of SBP in nighttime were significantly higher in the total cSVD burden progression group. The DBP SD and CV during daytime were significantly higher in the patients with total cSVD burden progression, but the SD and CV of DBP during 24-h and nighttime did not differ significantly between two groups. Both the SBP wSD and the DBP wSD were significantly higher in the progression group than those in the non-progression group (Table 5). Considering the ceiling effect for the patients with total 5 markers in assessing the progression, we excluded the 4 patients with total score of 5 and did the binary logistic regression in the left 136 patients. Binary logistic regression analysis revealed that SBP SD in daytime was a significant predictor for total cSVD burden progression [OR = 2.732, 95% CI = 1.150–6.490 (per 5 mmHg increase in SD), *P* = 0.023], but after adjusting for age, sex, blood pressure levels, the baseline cSVD score and other conventional risk factors (smoking, diabetes mellitus, hyperlipidemia, and BMI), the prediction of higher SBP SD for total cSVD burden progression was not significant (OR = 2.752, 95% CI = 0.984–7.692 (per 5 mmHg increase in SD), *P* = 0.054). While wSD was taken as the BPV metrics into the regression analysis, SBP wSD was found an independent risk factor for total cSVD burden progression [OR = 2.577, 95% CI = 1.103–6.019 (per 5 mmHg increase in SBP wSD), *P* = 0.029], after adjusting the factors above, the result was still significant [OR = 2.990, 95% CI = 1.053–8.496 (per 5 mmHg increase in SBP wSD), *P* = 0.040]. To the contrast, the DBP wSD was not showed an independent risk factor in the cSVD burden progression whether or not the other factors were adjusted.

## Discussion

Our research is a retrospective study to explore the relationship of 24-h BPV and the total MRI cSVD burden, and the impact of BPV on total cSVD burden progression in inpatients with cerebrovascular disease. We found that SBP levels in all period of day increased significantly with greater total cSVD burden on MRI and greater SD and CV of both SBP and DBP during 24-h and daytime as well as SBP wSD in patients with greater total cSVD burden. Besides, our study found higher SBP levels, greater SBP SD and CV during 24-h, daytime and SBP SD during nighttime, greater DBP SD and CV during daytime, greater SBP wSD and DBP wSD in patients with the total cSVD burden progressed. Logistic regression analysis showed that SBP SD in daytime and SBP wSD were independent risk factors for total cSVD burden and SBP wSD a significant predictor for total cSVD burden progression after adjusting for age, sex and other conventional risk factors. We use the ABPM to get the information of the blood pressure level and the 24-h BPV, for former studies have found 24-h ABPM is more efficient to predict the vascular risk than the clinic blood pressure levels (24–26). There were several metrics to calculate the BPV such as SD, CV, variation independent of mean (VIM), weighted SD (wSD), and the average real variability (ARV), but now there have been not widely accepted metrices or cutoffs. In our study we chose the most convenient and widely used metrics SD and CV to calculate the BPV. Besides, wSD was also used for it was considered to avoid the contribution of nocturnal BP fall to the BPV and correlate better with end-organ damage (27).

Several studies have investigated the relationship between BPV and individual cSVD markers, including lacunar infarcts, white matter lesion, EPVS and microbleeds, which identified higher BPV related to more severe cSVD markers (12–14, 28, 29). However, it is not uncommon that multiple cSVD markers are found simultaneously in a single patient and all the markers often share the same etiology and pathogenesis, thus total cSVD score comprising different markers has been used by different teams to represent the overall cSVD severity (4, 5, 15). Klarenbeek et al. (4) found higher SBP and DBP levels significantly related to total cSVD burden in patients with first episode of lacunar infarction. Yang et al. (15) took all the five cSVD markers into account and reported higher SBP level and greater SBP variability were significantly associated with greater total cSVD burden in population for physical examinations. Referring to the neuroimaging standards for research into small vessel disease (2, 3), we included all the neuroimaging markers into our score system and found SBP levels, the SD and CV of both SBP and DBP during 24-h and daytime as well as the SBP wSD correlated significantly with greater total cSVD burden in inpatients with cerebrovascular disease.

The longitudinal studies regarding cSVD were quite limited due to the difficulties in long-term follow-up given that cSVD is a slowly progressive disease. In CASISP study (14), higher baseline SBP and DBP variability was associated with increased deep brain microbleeds during 1–1.5 years follow-up. Yamaguchi et al. (30) found increased SBP and DBP SD were relevant to lacunar infarct and WMH development as well as cognitive function decline in Japanese community-based elderly people. In a large population-based study of TIA/stroke patients, premorbid SBP level showed a close relationship with cSVD burden, indicating a latent effect of SBP on total cSVD burden (31). Coincidently, we identified SBP SD during daytime an SBP wSD as significant risk factors for total cSVD burden and SBP wSD as a significant predictor for the cSVD progression in the present study, while other BPV metrics were not. Similar results were reported in other studies about BPV and cSVD as well as other target organ damage (15, 28, 32), probably because SBP has more impact on vascular risk including cSVD (33–35). In addition, different BPV metrics in different time period may not manifest the same aspects of blood pressure and may relate differently to specific target prognosis (35, 36).

Different from the previous studies, 17.1% patients had no markers of the cSVD, which was lower than that in studies of Yang, Staal, and Yang et al. (5, 15, 37). In the present study, the percentage of patients having all the five markers was 2.9%, higher than that of Yang's study (1.59%), but lower than that in studies of Staal (4%) and Klarenbeek et al. (4.9%) (4). The main reason for this inconsistency was the difference existed in study population. Contrast to the subjects for physical examination without symptom in study of Yang et al. (15), we recruited inpatients with cerebrovascular disease in the present study. In addition, recent small subcortical infarct and lacunar infarct were considered as one single cSVD marker in the present study for these two neuroimaging manifestations shared the same pathological and pathogenesis features. We also took brain atrophy into consideration since brain atrophy was found to be result of the hypertension target organ lesion and mediated the cognitive function decline caused by cSVD (23, 38, 39).

Although it was believed that traditional vascular risk factors, such as male, smoking, diabetes mellitus, high low-density-lipoprotein cholesterol level were associated with cerebrovascular events and cSVD (20, 28, 40–43), there was no statistical difference of these risk factors among different cSVD score groups, or between the cSVD progression and no-progression group in our study. Our finding was consistent with the results in former studies (4, 15, 44, 45). This may be due to the limited size of study population and the lack of detailed quantitative assessment of these factors, such as the amount of cigarettes and the level of glucose for longer time.

The underlying pathological mechanisms between BPV and cSVD burden have not been completely understood. Higher BPV has been considered to increase the stress on the vessel walls and lead to arterial stiffness and endothelial injury (1, 2, 46). The endothelial injury may increase the permeability of small vessels, finally resulting in cSVD development. Moreover, higher BPV often leads to sudden lowering of the BP and consequently causes decrease of cerebral perfusion, which is thought to be one of the pathogenesis mechanisms in cSVD (26).

There are some limitations in the present study. Firstly, we chose the inpatients who suffered from cerebrovascular disease and finished the reexamination of MRI after discharge. As a result, we may enroll the patients who were relatively more severe, which may lead to a selection bias in the study population. Moreover, there was heterogeneity in the subjects enrolled for several different major initial diagnosis. However, although having different diagnosis, the patients above were all with cerebrovascular disease generally and had common cerebrovascular risk factors such as diabetes, hypertension, smoking, which means we studied a group of patients with great risk of cSVD. Secondly, all the patients underwent the 24-h ABPM in hospital, which could not represent the true BP levels during their daily activity, The baseline 24-h ABPV could not also predict the long-term BP levels and BPV in the next years completely. Thirdly, the method of scoring total cSVD burden and its progression need to be modified for the following several reasons: Different field strength affected the judgment for microbleeds. Here we have both 1.5T and 3.0T MRI used and that may lead to some misjudgment. To handle that, the sequence of SWI was used to increase the sensitivity for microbleeds. Although atrophy was considered one of the cSVD markers, there was no widely accepted method and cutoff to assess it up to now. We evaluated brain atrophy according to the visual rating scale (22, 23) which was easily to use in daily clinical work but lack of precision. The total cSVD score is a semi-quantitative method and it would lead to the underestimation of the progression, for example, the patients with 5 scores at baseline would not get the score increase even though the cSVD actually progressed. To remove the ceiling effect, in the regression analysis for progression we excluded the 4 patients with 5 scores at baseline, but there were still bias and underestimation. Although the same method with ours was reported to judge cSVD progression (47), more investigation is needed to increase the objectivity and precision of cSVD assessment. Finally, given that our study was a retrospective study performed in a single center, the causal-effect relationship between the baseline BPV and the total cSVD burden and its progression in later years cannot be obtained, which needs further exploration in multi-center prospective and randomized clinical trials.

## Conclusions

Higher BPV were significantly related with total cSVD burden in inpatients with cerebrovascular disease and SBP SD during daytime as well as SBP wSD were independent risk factors for total cSVD burden. Besides, SBP wSD was an predictive factor for cSVD progression.

## Data Availability Statement

All datasets generated for this study are included in the article/supplementary material.

## Ethics Statement

This study was approved by the ethics committee of Peking University People's Hospital and all the patients provided written informed consent.

## Author Contributions

YF collected, analyzed and interpreted the patient data, and was a major contributor in writing the manuscript. CH revised the manuscript and helped to interpreted the data. LP and XG made contributions to the acquisition of data. YX designed the present study. All authors have read and approved the final version of this manuscript.

## Conflict of Interest

The authors declare that the research was conducted in the absence of any commercial or financial relationships that could be construed as a potential conflict of interest.

## Abbreviations

MRI: magnetic resonance imaging
cSVD: cerebral small vessel disease
BPV: blood pressure variability
ABPM: ambulatory blood pressure monitoring
SD: standard deviation
CV: coefficient of variation
SBP: systolic blood pressure
DBP: diastolic blood pressure.
